# Algorithms for left atrial wall segmentation and thickness – Evaluation on an open-source CT and MRI image database

**DOI:** 10.1016/j.media.2018.08.004

**Published:** 2018-12

**Authors:** Rashed Karim, Lauren-Emma Blake, Jiro Inoue, Qian Tao, Shuman Jia, R. James Housden, Pranav Bhagirath, Jean-Luc Duval, Marta Varela, Jonathan Behar, Loïc Cadour, Rob J. van der Geest, Hubert Cochet, Maria Drangova, Maxime Sermesant, Reza Razavi, Oleg Aslanidi, Ronak Rajani, Kawal Rhode

**Affiliations:** a School of Biomedical Engineering & Imaging Sciences, King’s College London, UK; b Epione, INRIA Sophia Antipolis, Nice, France; c Department of Cardiology, Haga Teaching Hospital, The Netherlands; d Leiden University Medical Center, Leiden, The Netherlands; e Robarts Research Institute, University of Western Ontario, Canada; f IHU Liryc, University of Bordeaux, Pessac, France

**Keywords:** Left atrium, Left atrial wall thickness, Myocardium, Atrial fibrillation

## Abstract

Structural changes to the wall of the left atrium are known to occur with conditions that predispose to Atrial fibrillation. Imaging studies have demonstrated that these changes may be detected non-invasively. An important indicator of this structural change is the wall’s thickness. Present studies have commonly measured the wall thickness at few discrete locations. Dense measurements with computer algorithms may be possible on cardiac scans of Computed Tomography (CT) and Magnetic Resonance Imaging (MRI). The task is challenging as the atrial wall is a thin tissue and the imaging resolution is a limiting factor. It is unclear how accurate algorithms may get and how they compare in this new emerging area. We approached this problem of comparability with the Segmentation of Left Atrial Wall for Thickness (SLAWT) challenge organised in conjunction with MICCAI 2016 conference. This manuscript presents the algorithms that had participated and evaluation strategies for comparing them on the challenge image database that is now open-source.

The image database consisted of cardiac CT (n=10) and MRI (n=10) of healthy and diseased subjects. A total of 6 algorithms were evaluated with different metrics, with 3 algorithms in each modality. Segmentation of the wall with algorithms was found to be feasible in both modalities. There was generally a lack of accuracy in the algorithms and inter-rater differences showed that algorithms could do better. Benchmarks were determined and algorithms were ranked to allow future algorithms to be ranked alongside the *state-of-the-art* techniques presented in this work. A mean atlas was also constructed from both modalities to illustrate the variation in thickness within this small cohort.

## Highlights

• An open-source atrial wall thickness CT and MRI dataset (n=20) with consensus ground truth obtained with statistical estimation from expert delineation (n=2).
• Exploring a range of metrics for evaluating and ranking wall segmentation and thickness algorithms (n=6), and benchmarks were set on each metric.
• New three-dimensional mean thickness atlases for atrial wall thickness derived from the consensus ground truth. The atlas was also transformed into a two-dimensional flat map of thickness.

## Graphical abstract

**Figure:**
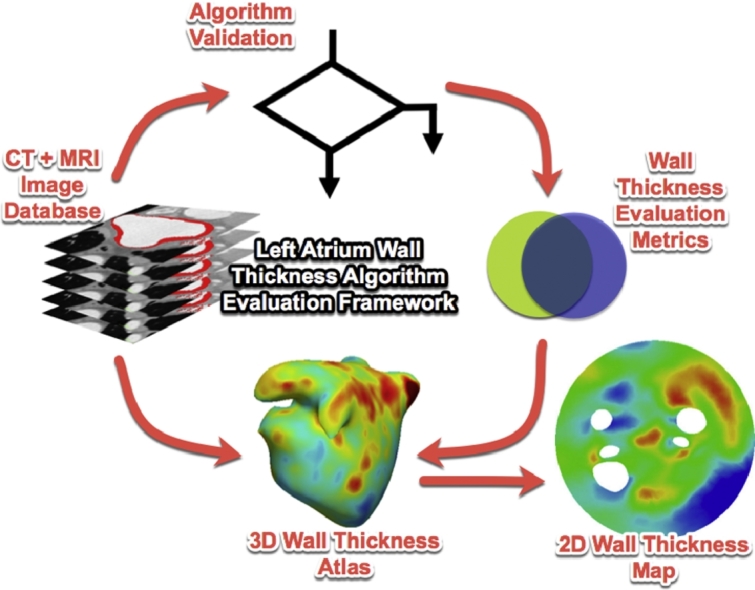

## 1 Introduction

In the past decade, algorithms for medical image analysis have grown rapidly with the availability of several open-source image processing and visualisation libraries. However, translation of these algorithms to the clinical environment has been limited despite their rapid development. Algorithms are usually validated in-house extensively, but it often remains unclear how they compare to other existing algorithms. Cross comparing the algorithm’s performance becomes a challenge with the absence of a common pool of data. It limits algorithm translation into the clinical workflow as a proper validation involves many challenges.

In recent years, there has been a rapid rise in open source datasets. A good example are the Kaggle data science challenges (www.kaggle.com). Data enrichment is essential for the latest generation of Big Data algorithms. Within the medical image processing community, several data segmentation challenges have been organised at conferences and meetings, each with its own unique theme. These have provided open source medical image datasets to the research community on which algorithms can be benchmarked.

Benchmarking is an excellent means of providing a fair test-bed for comparing algorithms. There exists an index of past challenges within the medical image processing and it can be found on the Cardiac Atlas project page in https://www.cardiacatlas.org/web/guest/challenges. In the cardiovascular imaging domain, some recent challenges for establishing benchmarks include left atrial fibrosis and scar segmentation (Karim et al., 2013), left ventricle infarction (Karim et al., 2016), cardiac motion tracking (Tobon-Gomez et al., 2013) and coronary artery stenosis detection (Kirisli et al., 2013).

### 1.1 Motivation

Atrial fibrillation (AFib) is the commonest cardiac arrhythmia globally, affecting 1.0–1.5% of the general population. As its prevalence is higher in older patients, it is likely to become even more common as the population ages, potentially leading to what some have called an ‘epidemiological time bomb’, with increasing numbers of patients being diagnosed and needing treatment (Lip and Tse, 2007).

In certain patients, AFib may not respond to treatment (drug resistant AFib), or may return after a period of treatment (drug refractory atrial fibrillation). In these patients, catheter ablation may be used to remove and destroy areas of the heart wall where ectopic foci are. These are regions of the atrium that sustain irregular rhythms in fibrillation. Lesions are created by ablating and scarring the ectopic areas. The amount and extent of scarring is important. The study in Arujuna et al. (2012) showed that the proportion of scar and edema can be used to predict outcomes of AFib ablation procedures.

Many past studies including Arujuna et al. (2012) have analysed scar as two-dimensional. The thickness of scar is becoming more relevant as it is now understood that ectopic activity can prevail in scar that is *non-transmural* (Ranjan, Kato, Zviman, Dickfeld, Roguin, Berger, Tomaselli, Halperin, 2011, McGann, Kholmovski, Oakes, Blauer, Daccarett, Segerson, Airey, Akoum, Fish, Badger, et al., 2008). To measure transmurality of scar, left atrial wall thickness (LAWT) is an important prerequisite. Moreover, research into wall thickening are still in early stages and it is not clearly understood whether changes in wall thickness are caused by the disease; a predisposing factor in its development; or whether these changes and disease evolution are correlated to additional factors such as age, medical history or other cardiac disease (Dewland, Wintermark, Vaysman, Smith, Tong, Vittinghoff, Marcus, 2013, Whitaker, Rajani, Chubb, Gabrawi, Varela, Wright, Niederer, O’Neill, 2016). Having techniques and algorithms that can identify subtle wall thickening changes from cardiac imaging data can thus have many important applications and improve our understanding of wall thickening and AFib.

### 1.2 State-of-the-art for left atrial wall thickness

The problem of measuring LAWT is two-fold. Firstly, a segmentation of the wall from neighbouring structures is necessary. Secondly, the thickness should be calculated between the inner and outer walls of the segmentation. Some regions in the wall can have multiple solutions (Bishop et al., 2016). Also, the inherent thinness of the atrial wall makes its segmentation and thickness measurement complex and challenging. The atrial wall can have sub-millimetre thickness in some sections (Dewland et al., 2013) and this makes imaging of the wall and methods to measure thickness quite challenging.

At sub-millimetre thicknesses, the wall is captured in only a few pixels of the image. The gold-standard for measuring *in-vivo* thickness remains to be Computed Tomography (CT) as it can image the heart at sub-millimetre resolutions. In recent years there have been a few studies measuring LAWT with Magnetic Resonance Imaging (MRI). Although MRI does yet not provide the spatial resolution necessary, it can become the modality of choice as it is widely considered to be the gold-standard for assessing wall (myocardium) tissue viability.

A short review of existing techniques for measuring LAWT is summarised in Table 1. Researchers have attempted to measure wall thickness using various methods. Most methods rely on ruler-based measurements performed on 2D slices with digital callipers and without performing a prior segmentation of the wall. Only a few studies, such as Inoue et al. (2014) and Tao et al. (2016) propose advanced image analysis for segmenting the wall; both are validated in this work. Bishop et al. (2016) proposes constructing Laplacian field lines for measuring thickness from wall segmentations.

**Table 1:** Overview of previously published methods for wall quantification and segmentation.

Reference	Cohort	Method	Thickness sample	Important conclusions
Hall et al. (2006)	n=34 patients post-mortem with heart disease	Thickness measured at five sites in gross anatomical heart specimens: anterior wall, isthmus, posterior wall, septum, roof Hearts prepared using 10% formalin	Anterior wall: 1.86 ± 0.59 mm, Isthmus: 1.6 ± 0.48 mm, Posterior wall: 1.4 ± 0.46 mm, Roof: 1.06 ± 0.49 mm, Septum: 2.2 ± 0.82 mm	Roof was the thinnest area, with the septum the thickest, Men had higher average and maximum values at all sites, No significant relationship between wall thickness and age
Imada et al. (2007)	n=16 patients with CAF and n=17 patients with PAF	ECG-gated CT scans. Measurements taken at the anterior atrial wall by a trained observer	CAF group LAWT: 2.6 mm, PAF group was exactly the same as chronic group.	Similar degree of thickening between both disease cohorts. Extent of thickening linked to disease stage and time course
Hoffmeister et al. (2007)	n=26 patients with AFib and n=16 without AFib	CT scans	Mean LAWT: 2.4 ± 0.5 mm	LA has an increased volume and dimensions in AFib patients
Pan et al. (2008)	n=180 patients divided into different age groups	CT scans with measurements taken on the anterior and posterior walls	Mean LAWT in anterior wall: 2.0 ± 0.9 mm, 3.2 ± 0.2 mm and 3.7 ± 0.9 mm in 40–60, 60–80 and 80+ year olds. Mean LAWT in posterior wall: 0.7 ± 0.2 mm, 1.8 ± 0.2 mm and 2.4 ± 0.4 mm in 40–60, 60–80 and 80+ year olds.	Thickness of both anterior and posterior walls increased with age. The anterior wall was thicker than the posterior wall across all age cohorts
Platonov et al. (2008)	n=298 post-mortem autopsy	Measurements taken using callipers at three posterior wall locations: between the inferior pulmonary veins, centre and between the superior pulmonary veins	Between inferior pulmonary veins: 2.9 ± 1.3 mm. Between superior pulmonary veins: 2.3 ± 0.9 mm	Posterior wall thicker in patients with history of AFib
Nakamura et al. (2011)	n=186 patients separated into three groups: PAF, CAF and normal rhythm)	ECG-gated CT scans. LA wall thickness and volumes were calculated	CAF group: 2.1 ± 0.2 mm, PAF: 2.4 ± 0.2 mm, normal rhythm group: 1.9 ± 0.2 mm	Walls were thinner in patients with CAF than PAF. Wall thickening occurs before an increase in left atrial diameter: PAF and may have occurred due to the use of fresh specimens, rather than those fixed in formalin.
Beinart et al. (2011)	n=64 patients with AFib	CT scans measured at 12 locations: 3 roof sites, 3 floor sites, 4 posterior wall sites, 1 left lateral ridge site and 1 mitral isthmus site	Mean LAWT 1.89 ± 0.48 mm. Middle posterior wall: 1.43 ± 0.44 mm. Mitral isthmus: 2.05 ± 0.47 mm, Left lateral ridge: 2.10 ± 0.63 mm Middle superior posterior wall: 2.15 ± 0.74 mm	Variation between patients and between sites within the same patient. Roof was thicker than the floor and Isthmus thicker than the posterior wall. The left lateral ridge was thicker than most regions
Dewland et al. (2013)	n=98 patients with AFib and n=89 control patients	CT scans analysed by a computer algorithm and thickness measured at the inter-atrial septum, below right PV, atrial appendage and anterior wall	AFib group LAWT: 0.7 mm, and control group: 0.9 mm	Thinner atrial wall at all sites in AFib patients
Suenari et al. (2013)	n=54 patients with heart disease	From CT scans, the left atrial wall at various locations was measured manually	Differentiation in LAWT found in superior and inferior left lateral ridge	Left lateral ridge significantly thicker in a group of patients with recurring AFib
Hayashi et al. (2014)	n=34 patients with AFib and n=34 control patients	CT scans measured at 11 separate locations	Mean LAWT in AFib cohort: roof: 2.20 ± 0.51 mm, mid-posterior wall: 1.44 ± 0.17 mm, inferior-posterior wall: 1.64 ± 0.25 mm, Mitral Isthmus: 2.38 ± 0.36 mm	No significant differences in thickness between control and disease groups.
Hsing et al. (2014)	n=15 patients with AFib post-ablation	Measured from Gadolinium-weighted MR scans taken at different time-points: before ablation, 24 hours post-ablation and at 30 days post-ablation. Measurements made at a single site by a trained observer	Mean LAWT before ablation: 7.0 ± 1.8 mm, After ablation: 10.7 ± 4.1 mm	Increased atrial wall thickening was seen in the post-ablation scans: early wall thickening and swelling correlated to scar formation seen on the 30-day scan
Takahashi et al. (2015)	n=75 patients, and with heart disease (n=25)	From CT images, images pre-processed to remove wall fat	Anterior LAWT in diseased: 1.93 ± 0.44 mm and control: 1.65 ± 0.44 mm. Posterior LAWT in diseased: 1.93 ± 0.40 mm and control: 1.61 ± 0.31 mm	Thickening of the left atrial wall and PV junction in atrial fibrillation
Inoue et al. (2016)	n=86 patients with AFib	CT scans analysed by a computer algorithm using blood pool mesh vertex normal traversal. Thickness measured at 12 anatomical sites	Recurrent AFib group: 1.6 ± 0.6 mm, Non-recurrent AFib group: 1.5 ± 0.5 mm	Increased thickness has a small but significant effect on post-ablation recurrence and reconnection
Varela et al. (2017)	n=10 healthy volunteers, n=2 AFib patients	Novel MRI scan with 1.4 mm isotropic resolution and post-processed thickness maps using an average of nearest neighbours method for measuring cortical thickness	Healthy cohort thickness atlas: 2.7 ± 0.7 for right atrium and 2.4 ± 0.7 mm for left atrium. LAWT in Afib patients (n=2): 3.1 ± 1.3 mm and 2.6 ± 0.7 mm	MRI-based *in-vivo* measurement of atrial wall is agent-free and unique in literature. General agreement with previous CT studies.

Studies that measured the atrial wall thickness using an imaging modality are listed in chronological order. Abbreviations used: AFib – atrial fibrillation, PAF – paroxysmal atrial fibrillation, CAF – chronic atrial fibrillation, PV – pulmonary vein, LAWT – left atrial wall thickness.

In most existing methods, the measurements are sparse and performed only on a few selected locations on the atrial wall. For example, in Suenari et al. (2013), Beinart et al. (2011) and Nakamura et al. (2011) the measurements were on landmarked points; in Dewland et al. (2013) and Koppert et al. (2010) they were made only on axial planes. There is also lack of consistency of these chosen locations. For example, Takahashi et al. (2015) used 13 different points whilst Nakamura et al. (2011) used only a single area. Since wall thickness does vary with position (the left lateral ridge being an especially thick region, whilst the posterior wall usually has a lower thickness than the anterior wall), two studies which had the same patient cohort and measuring method could have vastly differing results if different measurement regions were chosen.

Another issue is that studies have different patient cohorts, and thus atrial wall thickness will vary. It is known that thickness varies with gender, age and disease status, including congenital abnormalities (Pan et al., 2008). Not all wall measurements involve in-vivo imaging. Some of the methods performed are *ex-vivo* on post-mortem hearts. The wall thickness can be reduced due to tissue preservation processes such as fixation and studies in Hall et al. (2006) and Wolf et al. (2009) have shown that this reduces thickness by 0.25–0.75 mm when compared to fresh specimens.

### 1.3 Proposed work

In this paper we propose a benchmark for future algorithms for segmenting and measuring LAWT from cardiac CT and MRI images. Measurement of LAWT is an important problem in cardiac image analysis. To demonstrate the benchmark, algorithms were evaluated on CT datasets (n=10) and MRI datasets (n=10) by comparing the consensus ground truth segmentation obtained from experienced observers. The segmentations were assessed with three different metrics: wall thickness, Dice metric and tissue volume/mass.

Algorithms evaluated in this paper are published works which were submitted as a response to the open challenge put forth to the medical imaging community at the Medical Image Computing and Computer Assisted Intervention (MICCAI) annual meeting’s workshop entitled Segmentation for Left Atrial Wall Thickness’ (SLAWT) data segmentation challenge. Each participant designed and implemented an algorithm which segmented the atrial wall in the CT dataset. There were no participants for the MRI dataset, and only standard image processing techniques could be evaluated. The datasets are now open-source and publicly available via the Cardiac Atlas project challenge website: http://stacom.cardiacatlas.org.

Also in this paper, we constructed a wall thickness atlas from the consensus ground truth in each dataset. The average thickness in different sections of the atrium was also calculated in this small cohort. It was demonstrated that the atlas could be used for predicting thickness in new cases using atlas propagation. A novel 2D flat map representation of the atlas was also computed. To our knowledge, this is the first left atrial wall thickness two- and three-dimensional atlas obtained using CT imaging data.

## 2 Methods

### 2.1 Image database

The image database consisted of CT images (n=10) from patients with cardiac diseases and MRI images (n=10) from healthy volunteers. The CT datasets consisted of four females with an age range of 43–77. The MRI dataset consisted of 3 females with an age range of 21–30. The images within each modality were obtained from a single centre. The imaging parameters are summarised in Table 2. The CT images are coronary CT angiography scans, with intravenous contrast agent injection. The scans were ECG-gated and acquired in a single breath hold. They were reconstructed to a 0.8 to 1 mm slice thickness, with a 0.4 mm slice increment and a 250 mm field of view. The image matrix was kept at a 512 × 512 matrix, constructed with a sharp reconstruction kernel. The MRI images were acquired in a Philips 3T Achieva scanner in a para-axial plane using a phase-sensitive inversion recovery sequence with a 3D FLASH readout, typical field-of-view (FOV): 280 × 190 × 120 mm, isotropic 1.40 mm acquisition resolution.

**Table 2:** Image acquisition.

	CT	MRI
Scanner type	Philips Achieva 256 iCT	Philips 3T Achieva
Sequence	Angiography with ECG-gated and single breath hold	3D FLASH, respiratory gating and acquired at mid atrial diastole
TE, TR, TI	–	2.7 ms, 5.9 ms, 450–700 ms
Voxel in-plane	0.8–1 mm	1.4 mm
Slice thickness	0.4 mm	1.4 mm

Image acquisition parameters for the challenge CT and MRI data. Abbreviations: TE - Echo time, TR - Repetition time, TI - Inversion time.

### 2.2 CT Algorithm 1: INRIA Sophia-Antipolis (INRIA) – Marker-controlled Geodesic active contours

#### 2.2.1 Background

Region-growing flood-fill is a well-known image processing technique which recursively aggregates all pixels that are connected to a seed pixel. The main limitation of region-growing is the leaking of regions into neighbouring structures. This limitation can be overcome by using a different class of methods known as deformable surfaces, which starts with an initial surface and deforms based on the new region (Terzopoulos, 1986, Montagnat, Delingette, Ayache, 2001). Leaks are prevented as the deforming surface is constrained to maintain its shape. A special case of deformable models known as Geodesic active contours (GAC) was used in this work (Caselles et al., 1997).

#### 2.2.2 Implementation

**Fig. 1:**
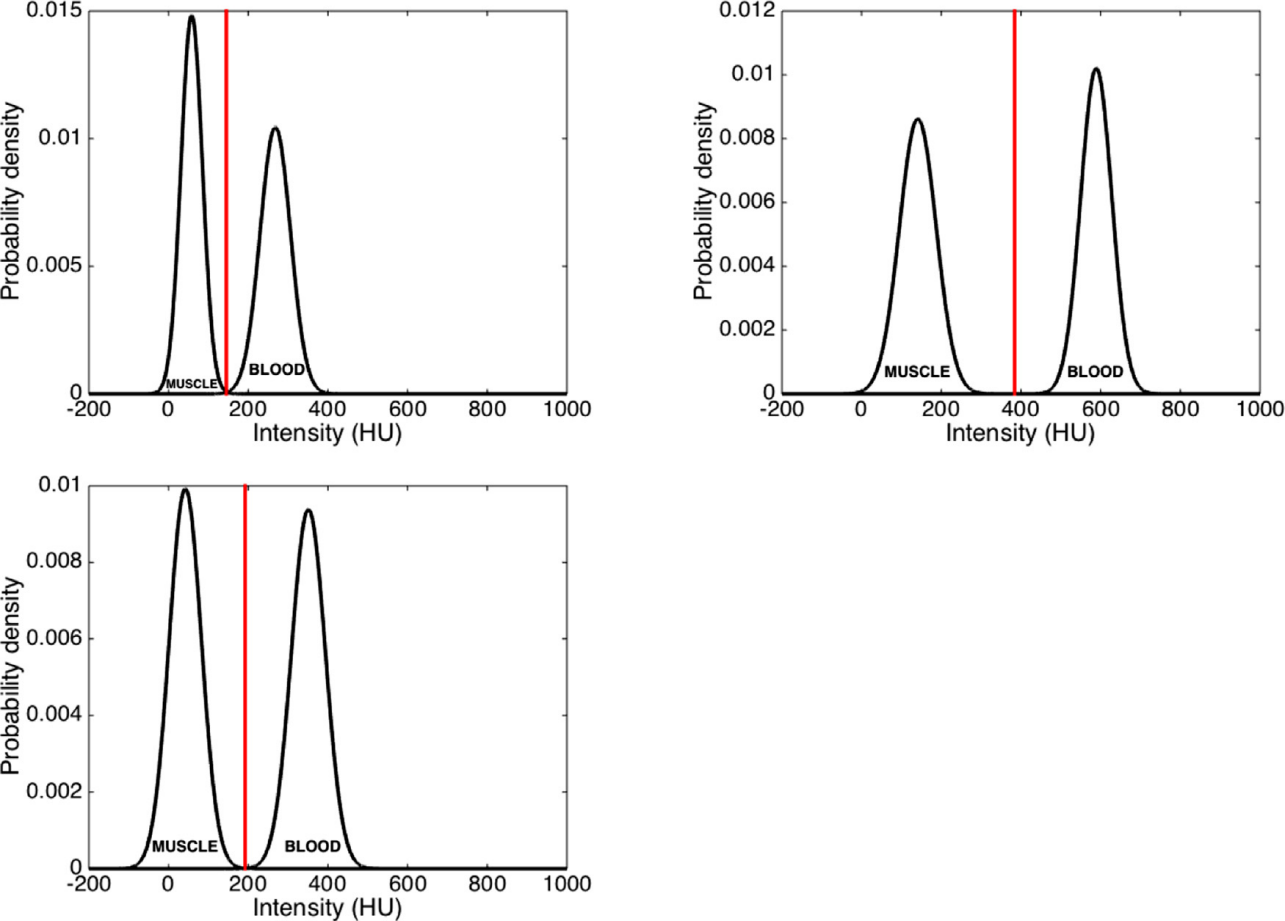
Investigating the thresholds calculated in the INRIA method using images from the database. The threshold calculated in three separate cases shown as a vertical line, together with Gaussian distribution best-fit models for blood and muscle tissue intensities.

Region growing was used to obtain the inner boundary of the atrial wall or endocardium. The endocardium acted as an initial contour for an ensuing GAC step. The GAC step then further expanded the endocardium to reach the outer boundary or epicardium. Region-growing was initiated from a seed voxel inside the blood pool and a threshold (*t_h_*) was calculated and obtained by sampling intensity distributions in the ventricular myocardium and atrial blood pool:

(1) $$t_{h}=\frac{\mu_{m}\sigma_{b}+\mu_{m}\sigma_{b}}{\sigma_{m}+\sigma_{p}}$$

where (*μ_m_, σ_m_*) and (*μ_b_, σ_b_*) are the mean and standard deviations of the intensity distributions for ventricle myocardium and atrial blood pool respectively. Calculations of this threshold value using Eq. (1) are illustrated in Fig. 1 in three randomly selected cases from the image database.

**Fig. 2:**
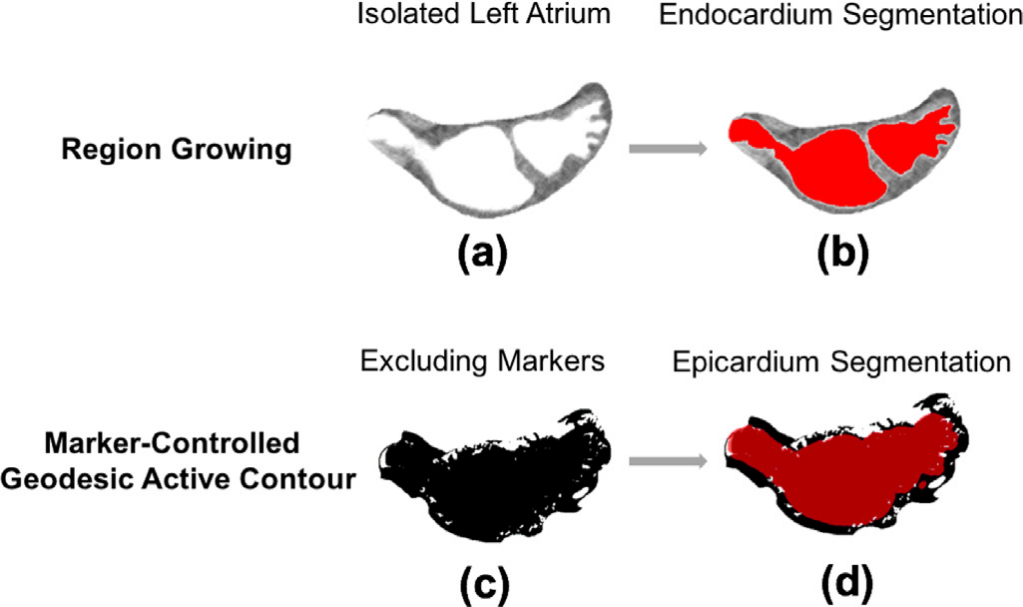
The intermediate steps in the INRIA method: (a) atrium and epicardium (i.e. wall) isolated from CT image, (b) region growing filling inner chamber, (c) inner chamber initialises active contour which expands into epicardium, (d) epicardium obtained from subtraction of region growing and active contour.

In the GAC step, the initial contour obtained from region growing was deformed to take the shape of the epicardium under the following conditions:

(2) $$\frac{\partial u}{\partial t}=g\left(c+\kappa \right)\left|\nabla u\right|+\nabla u\nabla g$$

where *u* is the GAC initial contour, *c* is a constant to provide a steady velocity for the deformation; *κ* is related to the curvature of the GAC to prevent leaks and avoid high curvatures, and *g* is an edge detector function of the image, which was strictly decreasing near the epicardium and 0 beyond the epicardium. In Eq. (2), *gc*|∇*u*| and *gκ*|∇*u*| are terms that relate to the progression and curvature of the deforming surface respectively. And finally, the term |∇*u*∇*g*| relates to the expansion of the surface. Please refer to Fig. 2 for an illustration of the steps involved in this process for extracting the epi- and endocardium for atrial wall.

### 2.3 CT Algorithm 2: Leiden University Medical Centre (LUMC) – Multi-atlas registration and level-set method

#### 2.3.1 Background

Atlases, which are labelled training images, lie at the core of atlas-guided segmentation methods. These methods have become one of the most-widely used successful segmentation techniques in biomedical images. Early atlas-guided segmentation methods were dominated by *probabilistic* atlas-based methods where only a single atlas was available and it encoded the probability of observing a particular label at a given location. The new image was segmented in the atlas coordinate frame with a probabilistic inference procedure that utilised a parametric statistical model. However, in recent times, multi-atlas segmentation methods have also become common. In a multi-atlas segmentation, each atlas is available for segmenting the new image using pair-wise registration between each atlas and the new image. The results from pair-wise registration are used to propagate the atlas labels to the new image, based on the most frequent label selected, also known as *majority voting*. A recent survey of multi-atlas segmentation methods can be found in Iglesias and Sabuncu (2015).

#### 2.3.2 Implementation

**Fig. 3:**
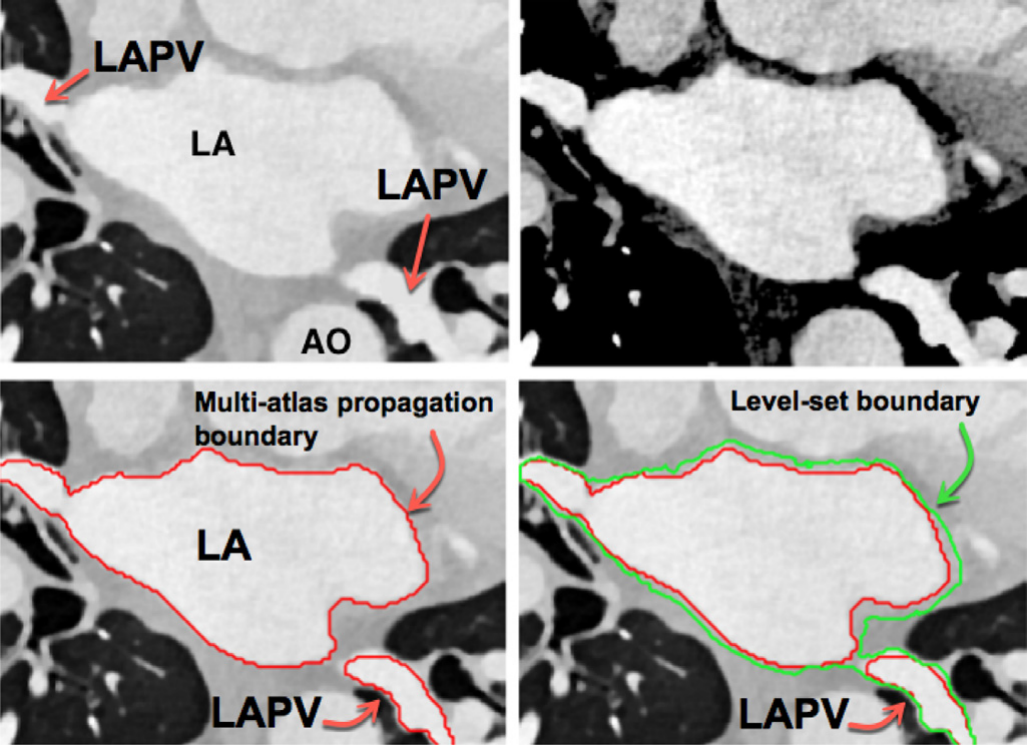
Intermediate steps in the LUMC method. Top row left to right: Original CTA, wall enhancement. Bottom row left to right: inner boundary (in red) obtained with multi-atlas propagation, outer boundary (in green) with the level-set operation. Abbreviations: LA - left atrium, AO - Aorta, LAPV - Pulmonary vein of the left atrium. (For interpretation of the references to colour in this figure legend, the reader is referred to the web version of this article.)

In this work, multi-atlas segmentation with majority voting was used for obtaining the inner boundary of the atrial wall. Ten individual atlases were used as described in Tao et al. (2016). The multi-atlas step determined the inner boundary. For the outer boundary, the atrial wall was first enhanced to mitigate the limited soft tissue contrast of atrial wall in CT Angiography (CTA) images. A non-linear transformation, such as square-root of the intensities, suppressed the high intensity signals due to blood. Using prior knowledge of tissue Hounsfield units (HU) for myocardial tissue (100–300 HU) and epicardial fat (−100 to −50 HU), a dynamic range of 0–400 HU was selected. This accounted for the partial voluming between the thin atrial wall, blood and low HU fat. Pixels within this selected dynamic range were enhanced with a square-root non-linear transformation. This resulted in clearer borders for obtaining the outer boundary. A level-set method was used to *advance and progress* the inner boundary to the outer boundary under an image force. The image force was defined as a combination of the image gradient and region information of the image that was non-linearly transformed. Fig. 3 shows images obtained at each step of the algorithm.

### 2.4 CT Algorithm 3: Robarts Research Institute (ROBI) – Blood pool mesh vertex normal traversal method

#### 2.4.1 Background

For analysing myocardial properties, the blood pool is a good initialising location. The blood pool intensities are normally homogenous in contrast enhanced scans, making its segmentation relatively straightforward. Once the blood pool is extracted, its surface can be utilised for exploring beyond blood pool, for example the myocardium. Some previous studies (Knowles, Caulfield, Cooklin, Rinaldi, Gill, Bostock, Razavi, Schaeffter, Rhode, 2010, Karim, Arujuna, Housden, Gill, Cliffe, Matharu, Rinaldi, O’Neill, Rueckert, Razavi, Schaeffter, Rhode, 2014) for detecting scar in myocardium have exploited the surface mesh of the blood pool for obtaining the maximum intensity along the mesh’s vertex normals. In this method, the blood pool mesh was obtained and a traversal of the mesh vertex normal was undertaken for computing the extent of the myocardial wall.

#### 2.4.2 Implementation

This method is an automated variant of the technique used to measure wall thickness from a AFib wall thickness study (Inoue et al., 2016). In this implementation, the LA blood pool and ventricular myocardium intensities are sampled using a paintbrush and two myocardial thresholds are calculated: an high intensity threshold between myocardium and blood pool is calculated as the mean of the myocardium and blood pool intensities. A low intensity threshold between the blood pool and surrounding tissue is calculated as two standard deviations below the mean myocardium intensity.

Using the above calculated intensities, the traversal distance is calculated along the vertex normal from each mesh vertex of the blood pool mesh. Along this normal ray, starting from the mesh vertex, the CT image was resampled at 0.1 mm intervals using trilinear interpolation, and each resampled point was classified based on the thresholds (blood pool, myocardium, or surrounding tissue).

The 3D position of the first resampled point along this ray that was classified as myocardium was defined as the initial estimate for the endocardial boundary. The 3D position of the first subsequent resampled point that was classified as surrounding tissue was defined as the initial estimate for the epicardial boundary. Based on the connectivity of the eroded mesh, two-neighbourhood averaging was performed twice for the endocardial boundary estimates and five times for the epicardial boundary estimates. The resulting points were defined as the final measurements for endocardial and epicardial boundaries, respectively. Traversal distance was defined as the geometric distance between the two boundaries, calculated on a point-by-point basis.

### 2.5 MRI Algorithms: Level-set methods, region growing and watershed segmentation

There were no participants for the MRI datasets and standard image processing techniques had to be implemented to establish a benchmark on these datasets. For the level-set method approach, the speed image was generated by firstly filtering noise with a median filter. This was followed with a gradient magnitude (GM) filter. The level-set was initialised from a segmentation of the endocardium with speed image as output of GM filter. The GM filter identified edges with sharp gradients near the epicardial borders. The level-set evolution halted at these borders. A simple subtraction of the level-set evolved image from the endocardium segmentation allowed the atrial wall to be obtained. An open-source implementation of level-set was used (Seg3D, SCI Institute, University of Utah, USA).

Region growing was also used to segment the MRI datasets. To remove noise and preserve epicardial boundaries, an anisotropic smoothing kernel was used (time step = 0.05, conductance = 0.5). The image was cropped to localise region growing and prevent leakage. Seed points were placed at various locations within the wall. The threshold was chosen selectively in each case and these generally ranged between 93 ± 9.4 and 125 ± 22.8 in the greyscale for lower and upper thresholds respectively. An open-source implementation of region-growing was used (ITKSnap, http://www.itksnap.org/)

Watershed segmentation (Roerdink and Meijster, 2000) was used to segment the MRI datasets. It was *marker-controlled*, utilising the image as a topographic surface and markers simulating the flooding from specific seed points. Noise was filtered with a median filter. Seed points were placed in the atrium and neighbouring structures (i.e. lungs, aorta, left ventricle). An open-source implementation of watershed segmentation was used (ImageJ, NIH, https://imagej.nih.gov).

### 2.6 Algorithm evaluation

#### 2.6.1 Reference standard: Label fusion

**Fig. 4:**
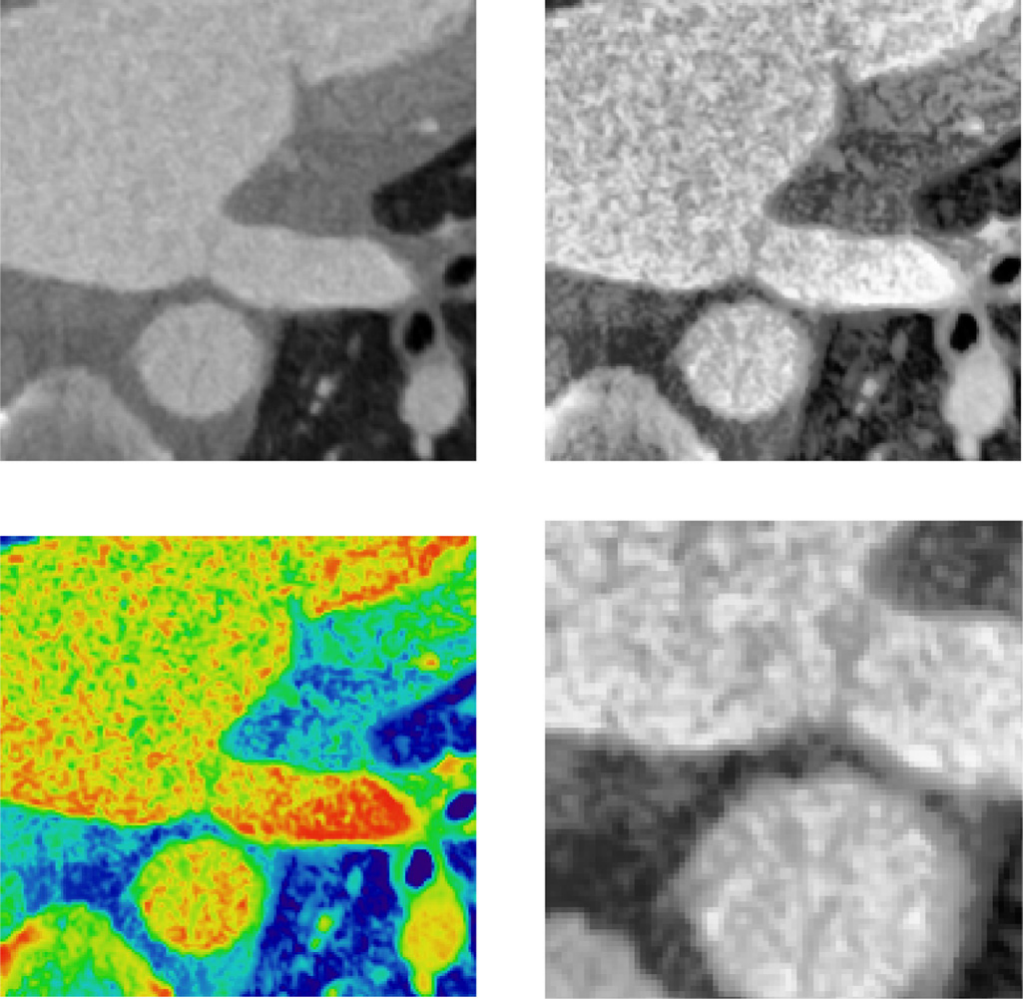
Contrast limited adaptive histogram equalisation (CLAHE) filter applied to each slice along with bilinear greyscale mapping for enhancing contrast in the wall.

A reference standard for atrial wall in each case was obtained as a consensus from two observers in both CT and MRI. The STAPLE algorithm (Warfield et al., 2004) performed simultaneous truth and performance level estimation calculating a weight for each observer, resulting in a consensus reference standard for each case. Both observers were experienced in cardiology scans, with one observer having several years’ experience in CT and working with patients suffering from ischaemic heart diseases. The CT in each case required image pre-processing prior to delineating the atrial wall. In some instances, it was necessary to enhance the appearance of the atrial wall using a Contrast Limited Adaptive Histogram Equalization (CLAHE) (Zuiderveld, 1994) step to allow the observer to accurately delineate it (see Fig. 4). In addition, for visualising the CT image on the display device and emphasising contrast in the atrial wall, the standard linear mapping for CT Hounsfield unit (HU) to greyscale was modified to a bilinear mapping to allow for the large CT dynamic range.

The atrial walls in the images were segmented as follows: (1) Each axial slice in the CTA was analysed separately, along with their orthogonal views. The high-contrast blood pool segmentation mask was available for each image and loaded as an overlay for contouring the epicardial border. (2) The left and right antra, roof, bottom, posterior and anterior aspects of the LA were identified and examined. Anatomic relationships between the esophagus and left PV, lungs and PV antrum were established for careful delineation of the wall. (3) The pixels belonging to the wall were labelled and noisy or dubious regions were excluded. (4) A single-pixel thick wall was included in regions where the wall could not be established by the observer. An image post-processing morphological dilation of the blood pool mask was used to achieve this. This ensured completeness of the atrial wall surrounding the LA.

In the MRI dataset, all three imaging planes were taken into consideration. In some slices, it was not possible to determine the border between the left atrium wall and the aortic root wall. In these instances, the entire border between the LA and the aortic root was included, as introducing such a separation in this region would be highly subjective.

#### 2.6.2 Evaluation metrics

Segmentations from each algorithm were compared with the reference standard for atrial wall. As no single metric is advocated as the best metric, three different types of metric were chosen for evaluating the segmentations. These were segmentation overlap, distance and volume-based measures, and they are briefly described below:

1. **Overlap metric:**
  The Dice overlap *D* is a metric for measuring the degree of overlap in segmentations. It calculates the proportion of true positives in the segmentation as follows:
  (3) $$D=\frac{2\times |T_{w}\cap G_{w}|}{|T_{w}\left|+\right|G_{w}|}$$
  where *T_w_* are pixels labelled as wall *w* in the *test* image by the algorithm, and *G_w_* are pixels labelled as wall in the consensus ground-truth segmentation.
2. **Distance-based metric:**
  The LAWT at every pixel location on the outer boundary of the wall was calculated in both the algorithm and consensus ground-truth segmentations. As wall segmentation contours from different algorithms and ground truth are bound to vary, they could not be compared at a pixel level. However, averaging them over slices enabled comparison at the slice level (i.e. for each slice). The thickness averaged over an entire slice or region *R* was used as a metric for assessing the accuracy of regional thickness from the segmentations. The regions considered were posterior and anterior sections of the LA. Additionally, individual slices in the LA axial orientation was also considered.
   The thickness *T_R_* of a region or slice was thus obtained by averaging the thickness over every pixel location *p_i_* ∈ *P* from the outer boundary of the segmented wall to the inner boundary *X*. The Euclidean distance *d*(.) between them was considered. The thickness *T_R_* was then given by:
  (4) $$T_{R}=\frac{\sum_{p}d(p_{i},X)}{P}$$
3. **Volume-based metric:**
  The total volume error between the algorithm’s output and the consensus ground-truth segmentation was measured. The total volume was calculated in each segmentation and converted into tissue mass (*M*) using the average human myocardial tissue density of 1.053 g/ml (Vinnakota and Bassingthwaighte, 2004). The difference in mass *ΔM* was noted between the volume in ground truth *V* and segmentation $\hat{V}$:
  (5) $$\Delta M=1.053\times |V-\hat{V}|$$

#### 2.6.3 Objective evaluation

An evaluation of how the algorithms handled artefacted regions in the images was important to understand whether they can be utilised in images of sub-optimal quality. In cardiac CT, excessive artefacts can be caused due to a number of reasons such as irregular heart beats, the inability to breath-hold, tachycardia and pacing wires or metallic valves (Roberts et al., 2008). Pacing leads and wires in the coronary arteries of patients who have undergone cardiac resynchronisation therapy (CRT) generate metallic streaks due to its titanium and platinum construction. The images used in this database were not free from artefacts, there was one image in the database with a CRT pacing wire and two images were of poor quality compared to the other images.

For objectively evaluating each algorithm, they were evaluated firstly on images of variable quality and secondly on slices with a pacing wire artefact. For variable quality evaluation, a number of slices were selected (n=237) from each image in the database and scored into one of the three categories: poor (n=68 slices), good (n=85) and excellent quality (n=84) by an observer experienced in cardiac CT scans. In each category, the LAWT measured by the algorithm and ground-truth were compared. A statistical measure known as *Pearson* Correlation coefficient (CC) was used to test and measure the linear dependence between LAWT measurements made by the algorithm and ground truth. CC also denoted by *ρ* is given by the covariance of two random variables (*A_i_*) and (*T_i_*) representing algorithm and ground-truth LAWT respectively:

(6) $$\rho \left(A,T\right)=\frac{1}{N-1}\sum_{i=1}^{N}\left(\frac{A_{i}-\mu_{A}}{\sigma_{A}}\right)\left(\frac{T_{i}-\mu_{T}}{\sigma_{T}}\right)$$

#### 2.6.4 Ranking

The evaluation metrics chosen could only provide isolated rankings. A ranking system was necessary for designing a fair and problem-specific challenge. There are a number of segmentation challenges in literature that provide a ranking schema. In the simplest of cases, metrics are evaluated independently and an isolated ranking assigned within each metric (Menze et al., 2015). There are others that compare the difference between expert segmentations and consider it to be the upper-limit or the 100% mark. One drawback is that it makes the assumption that expert segmentations are in very close agreement. A comprehensive ranking methodology can be found in Maier et al. (2017) and Murphy et al. (2011) where rankings are allocated on each metric and within each case. The final ranking is averaged over all metrics and cases, giving a comprehensive score for each algorithm so it may be ranked. In this work, the same approach as Maier et al. (2017) was adopted and each algorithm was assigned a ranking score and final rank.

### 2.7 Atlas of left atrial wall thickness

#### 2.7.1 Atlas construction with non-rigid registration

The consensus ground truth for wall segmentation was available for all images on the database. This allowed the construction of a LAWT atlas. The atlas creation comprised several steps. In the first step, 3D surface reconstruction of the left atrium was obtained using the marching cubes algorithm (Lorensen and Cline, 1987). The LAWT was calculated by projecting normals from each vertex on the 3D surface to the consensus wall segmentation. This generated 3D surface meshes containing the patient-specific LAWT. In the second step, the patient-specific mesh was registered to the mean left atrial anatomical shape using non-rigid registration, bringing the patient-specific LAWT to a common coordinate frame. In the third and final step, using data in the common frame, the mean LAWT, over all the datasets, could be calculated at every vertex location on the mean left atrium.

**Fig. 5:**
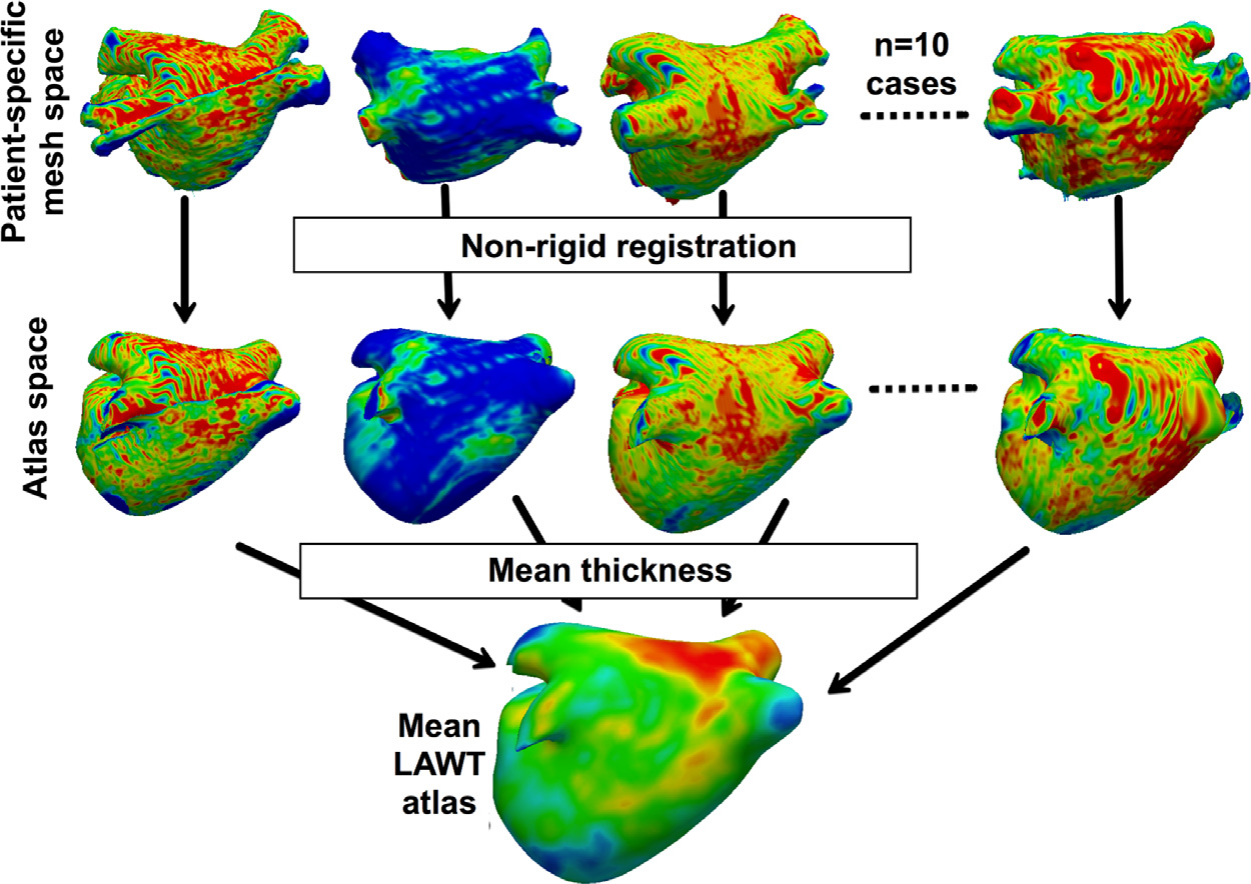
The steps involved in atlas construction. Meshes transformed from patient-specific space to a 4-vein anatomical atlas space with non-rigid registration. In atlas space, the thickness is averaged over all cases to generate the final LAWT atlas.

The atlas was represented on a mean shape. The mean shape was obtained by fusing four-vein anatomies in the benchmark datasets made available in Tobon-Gomez et al. (2015). A non-rigid registration was performed between each patient-specific LAWT surface mesh and the mean shape. The registration process comprised both a manual landmark selection step, followed by non-rigid registration of the two surfaces. The non-rigid transformation between two meshes used a free-form deformation between each vertex of the source mesh and the nearest target mesh vertex. The implementation in the Image Registration Toolkit (IRTK) was used (Schnabel et al., 2010). For an illustration of the atlas construction process on the mean shape please see Fig. 5.

#### 2.7.2 Atlas thickness propagation

The atlas could be used to predict thickness in new cases. This was demonstrated by registering the atlas to new cases and propagating thickness from the atlas to the new case. To validate this strategy, a leave-one-out (LOT) cross validation was performed on the image database. Ten separate atlases were constructed and each validated separately on the image that was excluded from the atlas. The validation involved a point-by-point analysis between the propagated LAWT values from the atlas and the actual LAWT obtained from the image. To propagate the LOT atlas thickness to each image, the LOT atlas was registered to the image that was excluded. The LAWT values from the atlas was propagated to the image using the nearest neighbour approach. The difference between the LAWT obtained from the LOT atlas and from the image was used to validate atlas thickness propagation.

#### 2.7.3 Flat thickness map

The mean LAWT atlas was obtained as a 3D surface with every vertex on the surface containing a mean thickness value. A flat 2D representation of the 3D atlas was also computed using a surface flattening and unfolding approach (Karim et al., 2014b). In the 2D representation, the whole atlas could be visualised simultaneously on a single plane. The atrium was divided into left, right, roof, anterior and posterior sections. The flat map representation was also sub-divided into the respective sections. The mean thickness in each section was determined and compared to values reported in the literature.

## 3 Results

**Fig. 6:**
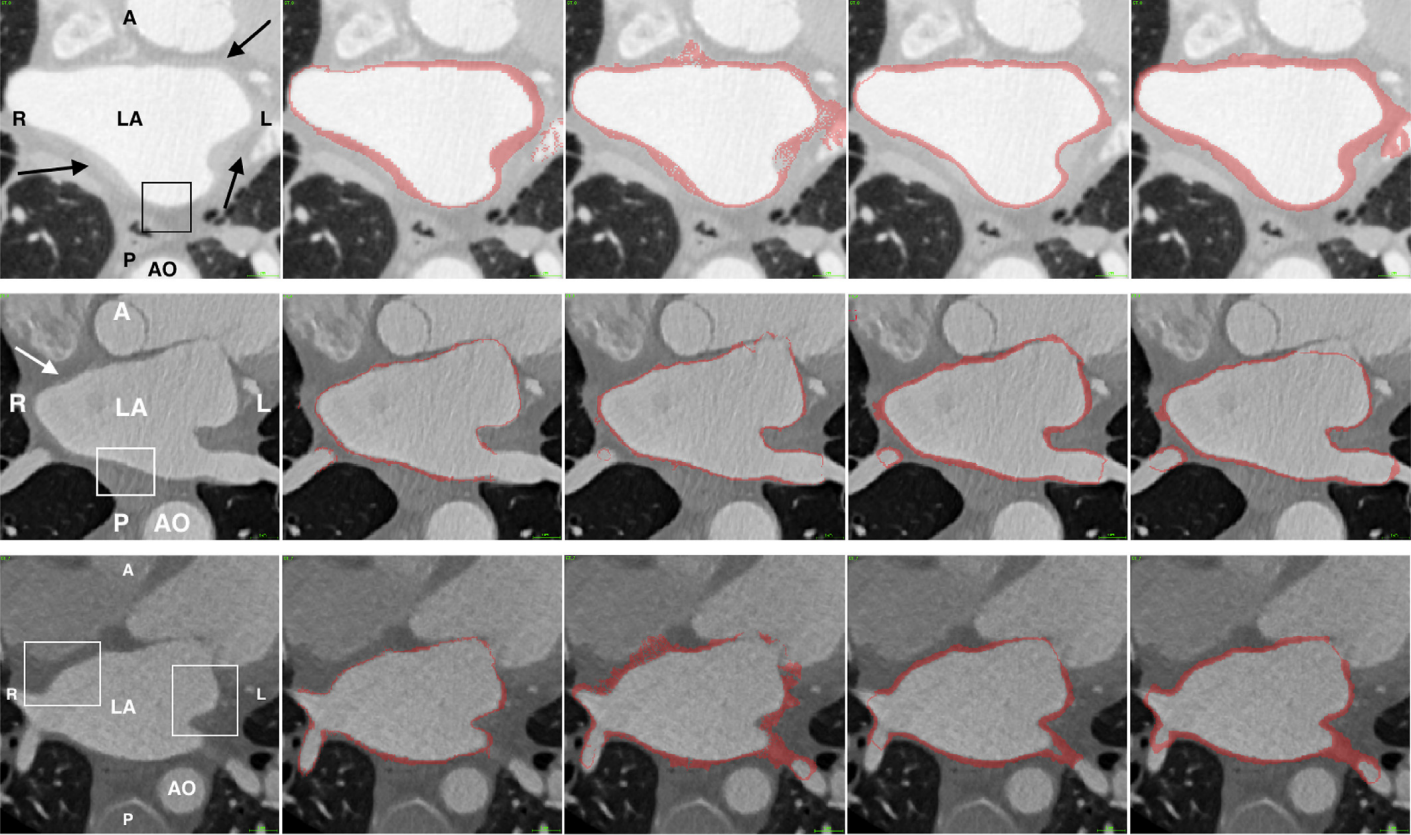
From the CT image database. Each row represents a separate case. Each column represents (from left to right): original CT, manual segmentation for ground truth, ROBI, LUMC and INRIA. Abbreviations: LA – left atrium, AO – aorta, R – right, L – left, A – anterior, P – posterior. The arrows indicate some regions where the wall has clear boundaries. The box highlights some regions where the wall boundaries are not clear.

**Fig. 7:**
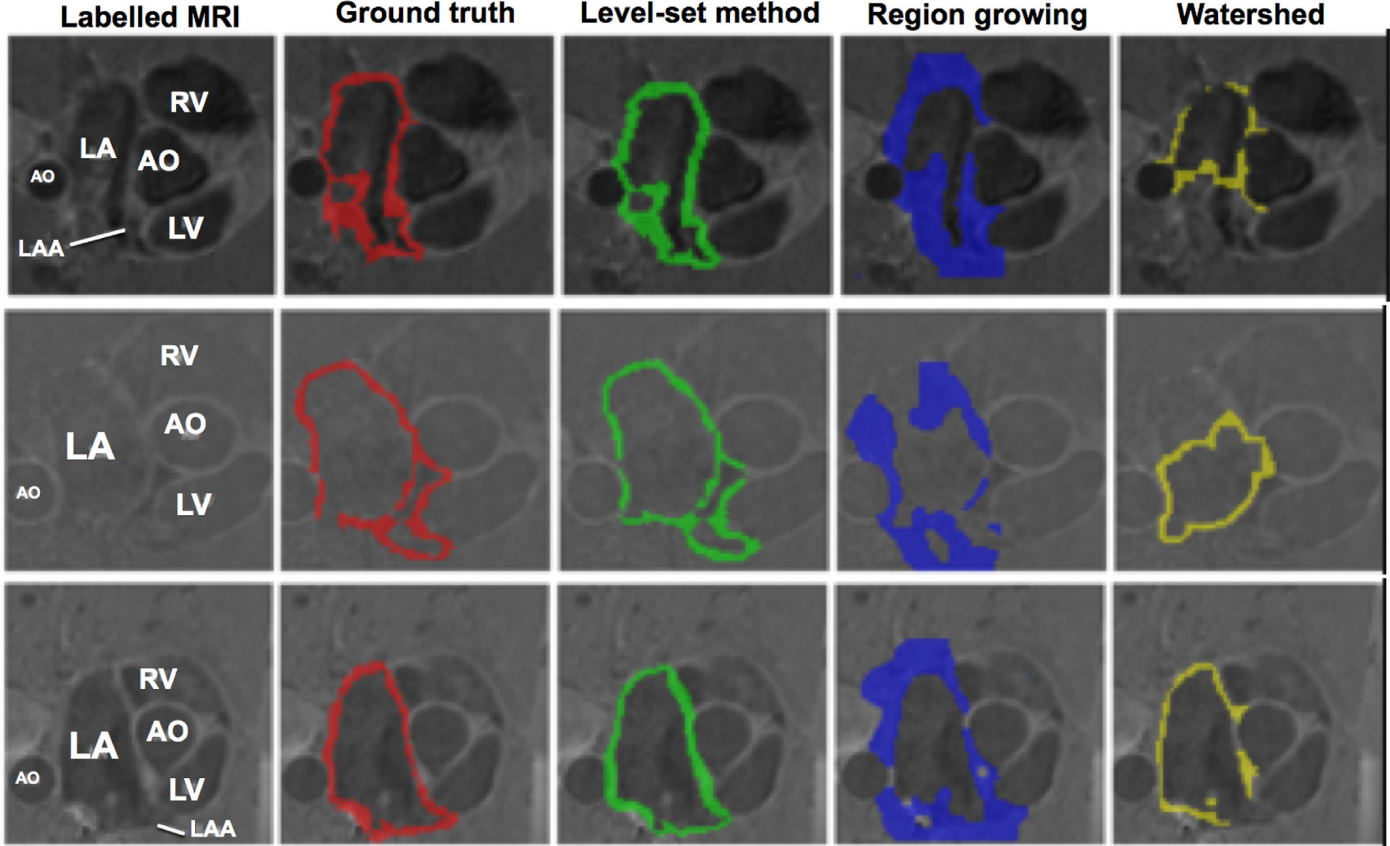
From the MRI image database. Each row represents a separate case. Each column represents (from left to right): original MRI, segmentation for ground truth, level-set method, region-growing and watershed segmentation. Abbreviations: LA – left atrium, AO – aorta, LV – left ventricle, RV – right ventricle, LAA – left atrial appendage.

The evaluated algorithms generated binary segmentations of the atrial wall from which the wall thickness could be derived. A sample of the segmentations obtained from the algorithms are illustrated in Fig. 6 for CT and Fig. 7 for MRI. The segmentations are analysed, compared and ranked in the following sections.

### 3.1 Algorithm segmentations compared to ground truth

The mean LAWT in the anterior section of wall as measured by ground truth was 1.16 ± 0.88 mm. This was obtained by averaging over all ten images, measured from approximately 19,800 locations on the LA in each image. In comparison, the mean LAWT in the anterior section as measured by the evaluated algorithms were 1.13 ± 1.02 mm, 1.34 ± 0.89 mm, 0.75 ± 0.38 mm for algorithms ROBI, LUMC and INRIA respectively. These were measured from approximately 22,400, 22,900 and 25,300 locations on the LA in segmentations in ROBI, LUMC and INRIA respectively.

**Fig. 8:**
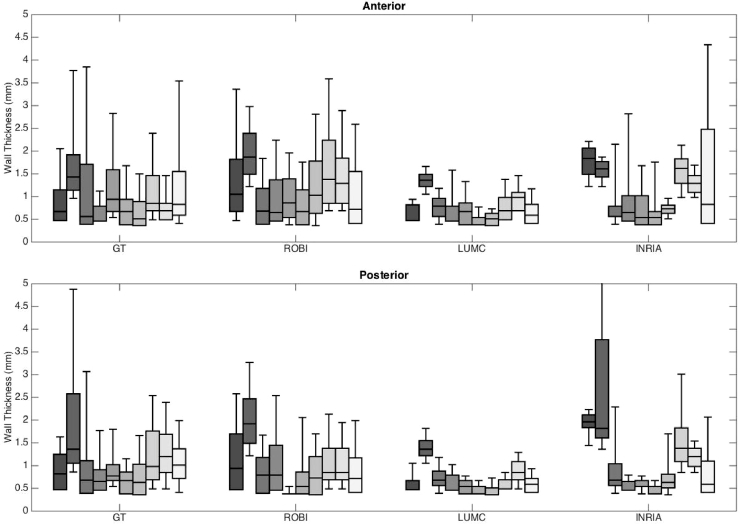
Comparison of the wall thickness in CT images (n=10) by algorithms (ROBI, LUMC and INRIA) and ground-truth segmentation (GT). The results are analysed with separate plots for anterior (top plot) and posterior (bottom plot) wall of the left atrium, using the same scale 0–5 mm to allow comparison. Wall thickness was averaged over each slice in the image.

The posterior section of the wall was analysed separately. The mean LAWT in the posterior section of all images measured by ground truth was 1.23 ± 1.10 mm, from an average of 19,120 locations on the LA in each image. The same posterior regions measured by the algorithms were 1.26 ± 0.83 mm, 0.78 ± 0.41 mm, 1.46 ± 1.57 mm by algorithms ROBI, LUMC and INRIA respectively. These were obtained by measuring LAWT from approximately 16,400, 14,700, 21,800 locations on the LA per image by algorithms ROBI, LUMC and INRIA respectively. These LAWT measurements made in both anterior and posterior sections are shown in Fig. 8. In this figure the LAWT distribution measured in each image is represented by boxes in the box-plots. The error in LAWT measurements was also quantified by taking the difference between the LAWT measured in ground truth and the algorithm, averaged over individual slices in the image and reported for each case. These errors are given in Table 3. The median error over all methods was 0.25 mm.

**Table 3:** Absolute error in wall thickness between algorithm and consensus ground truth. The error in millimetres was computed separately for the posterior (Post) and anterior (Ant) wall. The best result in each case is underlined for the anterior and posterior walls. The best overall are marked with an asterisk (*).

	ROBI		LUMC		INRIA	
	*Post*	*Ant*	*Post*	*Ant*	*Post*	*Ant*
Case 1	0.38	0.12	0.20	0.35	0.41	1.14
Case 2	0.44	0.56	0.07	0.00	1.05	0.46
Case 3	0.12	0.11	0.23	0.00	0.10	0.00
Case 4	0.19	0.14	0.00	0.19	0.29	0.19
Case 5	0.08	0.39	0.27	0.23	0.13	0.23
Case 6	0.00	0.13	0.29	0.29	0.03	0.13
Case 7	0.52	0.10	0.00	0.27	0.12	0.00
Case 8	0.53	0.13	0.16	0.49	0.93	0.40
Case 9	0.60	0.35	0.29	0.35	0.46	0.00
Case 10	0.11	0.29	0.24	0.42	0.83	0.42
Median	0.29	0.14*	0.22*	0.28	0.35	0.21
Inter-observer difference		Post = 0.25 mm,			Ant = 0.20 mm	

**Fig. 9:**
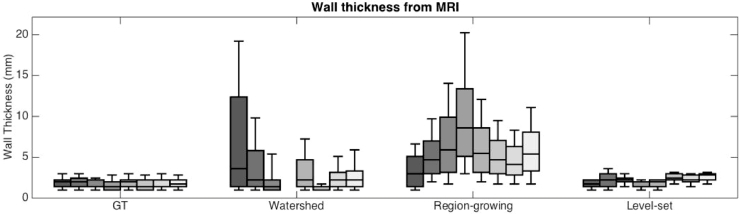
Comparison of the wall thickness in MRI images by algorithms and ground-truth segmentation. Bars represent distribution of wall thickness as measured within an image.

In the MRI datasets, a similar approach was undertaken to measure overall LAWT in each case. However, as the MRI resolution was lower than the CT, measurements were taken from an average of 2700 locations on the image. Furthermore, the analysis was *not* divided into anterior and posterior sections as in CT. The mean LAWT measured in MRI were 2.16 ± 0.58 mm, 6.04 ± 3.63 mm and 3.46 ± 3.57 mm in level-set, region growing and watershed algorithms. The full comparison of LAWT measured by these algorithms is given in Fig. 9.

**Fig. 10:**
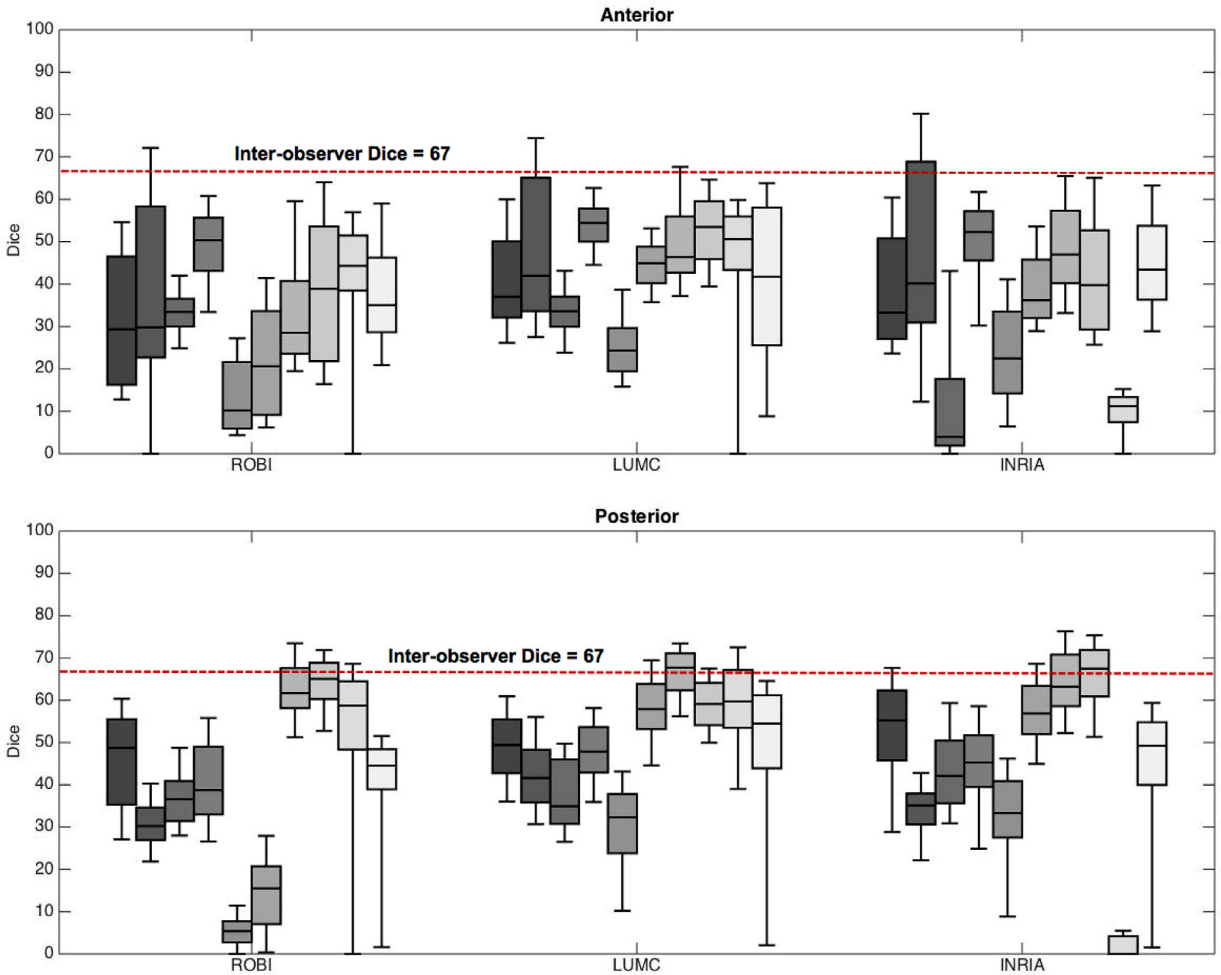
Segmentation overlap with ground truth assessed using the Dice overlap for each algorithm (ROBI, LUMC and INRIA). The DSI ranges from 0–100 with 0 indicating no overlap, and 100 indicating complete overlap. The median inter-observer Dice is noted within each plot.

The second metric for evaluating the algorithms was the degree of overlap between the algorithm and ground truth segmentations. This was measured using the Dice overlap metric. The values for Dice range between 0 and 100, with 100 representing a perfect overlap. The mean Dice overlaps in the anterior section of the LA were 33, 43 and 30 in ROBI, LUMC and INRIA respectively. These were obtained from an average of 136, 134 and 172 axial slices per image in ROBI, LUMC and INRIA respectively. In the posterior section of the wall, the Dice overlaps were found to be 39, 21 and 50 in ROBI, LUMC and INRIA respectively. These were again obtained from an average of 137, 136 and 135 axial slices per image in ROBI, LUMC and INRIA respectively. In Fig. 10, the Dice metric distribution in each image can be found in the box-plots.

In MRI, segmentations were evaluated similar to the CT dataset. The mean Dice over all slices was found to be 72, 39 and 67 for level-set, region-growing and watershed respectively. Level-set and watershed achieved far superior Dice overlap over the region growing and they were also statistically better (Wilcoxon signed rank *t*-test *p* < 0.05). Table 4 gives these Dice overlap results.

**Table 4:** Mean Dice overlap in the image processing algorithms tested on MRI datasets. Abbreviation - n/a – could not be computed.

	Level-Set	Region-Growing	Watershed
Case 1	85	44	81
Case 2	72	51	72
Case 5	68	35	64
Case 6	87	32	n/a
Case 7	78	36	61
Case 8	66	36	77
Case 9	73	48	67
Case 10	68	42	67
**Mean**	73	39	67
**Inter-observer Dice =**			56 ± 14

The final metric for comparison was volume-based. In this metric, the total volume of the segmentation was obtained from voxels in the wall segmentation. The total mass of atrial wall tissue could be calculated using the average human myocardial tissue mass density given by 1.053 g/ml. The total wall tissue mass from each segmentation and ground truth is given for each image in Table 5. In each case, to set benchmarks, the difference/error in the mass between the algorithm and ground truth segmentation was computed and averaged. The minimum and maximum mass difference/error were 3.84 g and 14.63 g respectively.

**Table 5:** Values shown are wall tissue mass (in grams) obtained using density as 1.053 g/ml. The last column notes the overall average for the mass difference ($\Delta \bar{M}$) between ground truth and each algorithm. The minimum and maximum differences are marked with an asterisk (*). The best result or closest approximation to ground truth in each case is underlined.

	GT	ROBI	LUMC	INRIA	$\Delta \bar{M}$
Case 1	19.33	35.69	30.53	30.00	12.62
Case 2	10.64	30.32	16.51	29.43	14.63*
Case 3	17.80	22.26	20.53	13.35	3.84*
Case 4	14.44	22.78	24.03	22.73	8.65
Case 5	13.97	20.02	27.44	20.17	8.49
Case 6	18.12	19.75	26.24	20.85	4.12
Case 7	13.99	29.75	17.74	23.91	9.71
Case 8	29.75	40.07	24.03	54.63	13.50
Case 9	20.26	30.02	20.66	34.38	8.01
Case 10	24.10	20.23	30.56	29.40	5.16
Inter-observer difference:				10.03 ± 4.0	

### 3.2 Inter-observer difference

Segmentations from the observers were compared on all metrics to determine a baseline within each metric. This baseline provided with two observations. Firstly, the agreement between the raters could be established providing an insight into how challenging the segmentation task was. Secondly, the baseline could be considered a limit above which an algorithm’s performance was deemed as excellent. The inter-observer Dice agreement was 67 ± 22 in CT, the difference in thickness was 0.25 mm and 0.20 mm for posterior and anterior walls respectively and the difference in volume was 10 ml. In MRI, the inter-observer Dice was 56 ± 14. Based on these values it was found to be a challenging segmentation task due to a generally thin wall structure of the atrium. Compared to other similar segmentation tasks, inter-observer Dice values of 70 ± 20 to 85 ± 8 are reported (Maier, Menze, von der Gablentz, Häni, Heinrich, Liebrand, Winzeck, Basit, Bentley, Chen, et al., 2017, Menze, Jakab, Bauer, Kalpathy-Cramer, Farahani, Kirby, Burren, Porz, Slotboom, Wiest, et al., 2015).

### 3.3 Leaderboard ranking

**Fig. 11:**
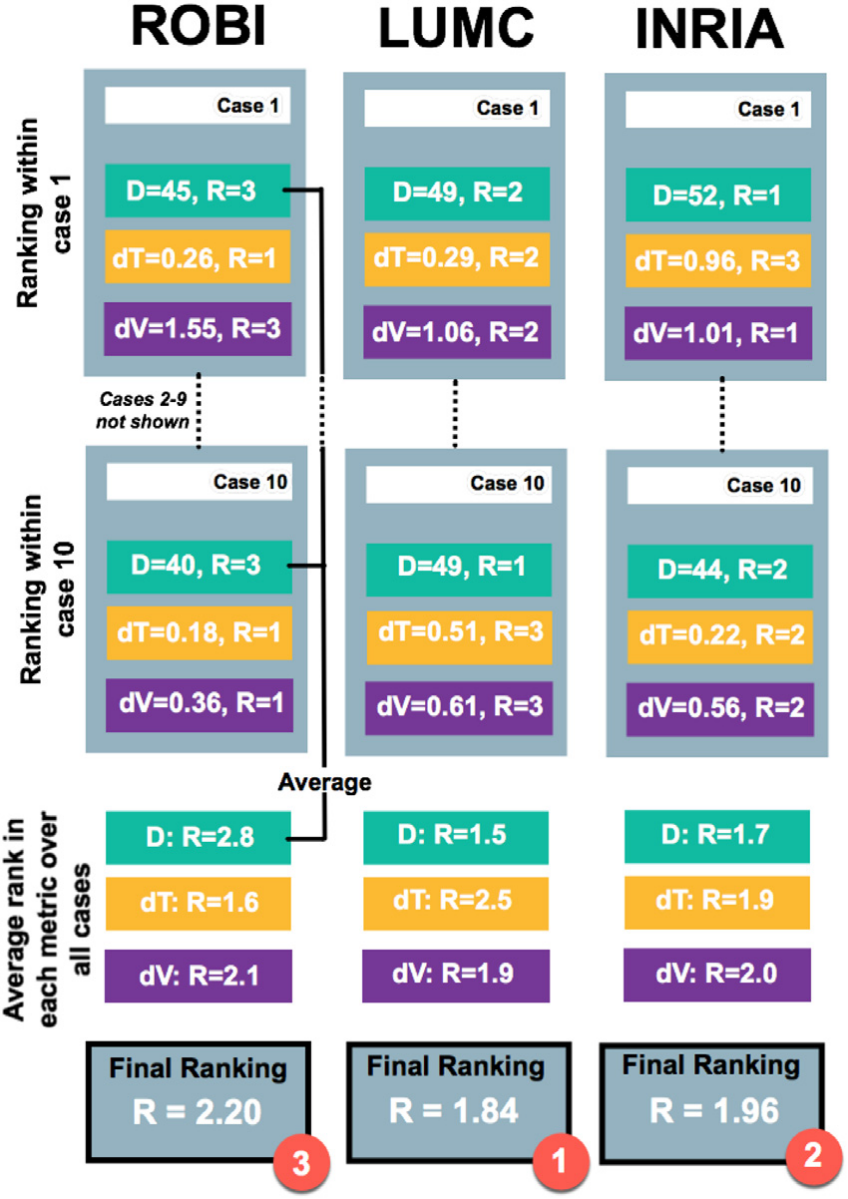
The ranking methodology illustrates how each algorithm was finally ranked in CT, based on its rank (*R*) within each metric: Dice (*D*), thickness (*dT*) and volume (*dV*).

The ranking methodology and final ranking of each evaluated algorithm was determined by averaging individual metric ranks for an algorithm over all cases. The final ranking and methodology has been illustrated in Fig. 11. The rankings within each metric are also listed in Table 6. CT Algorithms LUMC and INRIA achieved a close final ranking of 1.84 and 1.96 respectively. It was also observed that none of the algorithms consistently achieved a Dice agreement better than the human raters (i.e. 67). In the MRI dataset, the calculated rankings were: level-set=1.12, watershed=1.87 and region-growing=2.81, with level-set achieving the highest rank.

**Table 6:** Average ranking score within each metric culminating to a final score for each algorithm and the top rank is marked with an asterisk (*).

Metric	ROBI	LUMC	INRIA
Dice rank	2.85	1.32	1.90
Thickness rank	1.65	2.35	2.00
Volume rank	2.10	1.90	2.00
Final rank score	2.20	1.84*	1.96

### 3.4 Statistical analysis

A statistical analysis of the results was performed using the two-sided Wilcoxon signed-rank test (Wilcoxon, 1945), and the results are given in Table 7. It tests the null hypothesis that two related paired samples come from the same distribution. The lowest ranking algorithm in Dice was ROBI and the only statistical significance that was found with a confidence of 95% (*p* < 0.025) was in the Dice metric demonstrating that the Dice values were statistically poorer than the top-ranked LUMC. However, tests on other metrics (i.e. thickness and volume) showed that they were not statistically superior over one another. The final rank scores of the algorithms, although between 1 and 3, were close ( < 20%) with no clear winner in all three metrics. However, the statistical analysis concluded LUMC’s Dice scores were significantly better, which helped it achieve the top ranking score.

**Table 7:** *p*-values from test of statistical significance, with two-sided Wilcoxon signed-rank method, between algorithms for whether they are statistically superior or inferior over the other. *p*-values in bold indicate a significant difference with a confidence of 95% (*p* < 0.025 two-tailed).

Test	Dice	Thickness	Volume
ROBI / INRIA	0.386	0.444	0.721
ROBI / LUMC	**0.005**	0.721	0.284
LUMC / INRIA	**0.021**	0.798	0.332

### 3.5 Algorithm performance under variable imaging quality

Objective evaluation based on CT image quality demonstrated degradation of algorithm accuracy. The goodness of straight-line fit between actual and measured values with cross-correlation coefficients showed this trend. This trend in decrease of accuracy was in all algorithms. For top-ranked LUMC the goodness of fit decreased: ρ=0.92(excellentquality,ρ=0.56(good),ρ=0.19(poor). Table 8 lists them for other algorithms.

**Table 8:** Objective evaluation of each algorithm based on individually selected slices of differing quality (excellent, good and poor) and all slices combined. The statistical measures of correlation coefficient (*ρ*) and slope (*s*) were used to assess the algorithm’s accuracy. Values ρ=1 and s=1 are ideal.

	Excellent	Good	Poor	Combined
**ROBI**	ρ=0.64,s=0.25	ρ=0.59,s=0.30	ρ=−0.08,s=−0.05	ρ=0.51,s=0.25
**LUMC**	ρ=0.92,s=0.15	ρ=0.56,s=0.15	ρ=0.19,s=0.06	ρ=0.62,s=0.15
**INRIA**	ρ=0.96,s=0.85	ρ=0.67,s=0.70	ρ=0.26,s=0.25	ρ=0.73,s=0.75

**Fig. 12:**
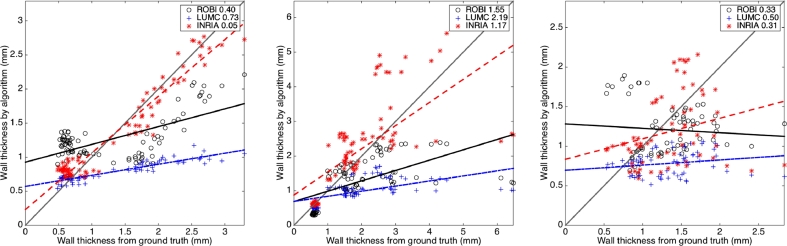
These demonstrate the reduction in accuracy of LAWT measurements with varying image quality. Each plot shows the MSE (in mm) between LAWT measured from ground truth and algorithm segmentations in images rated as excellent (left), good (middle) and poor (right).

**Fig. 13:**
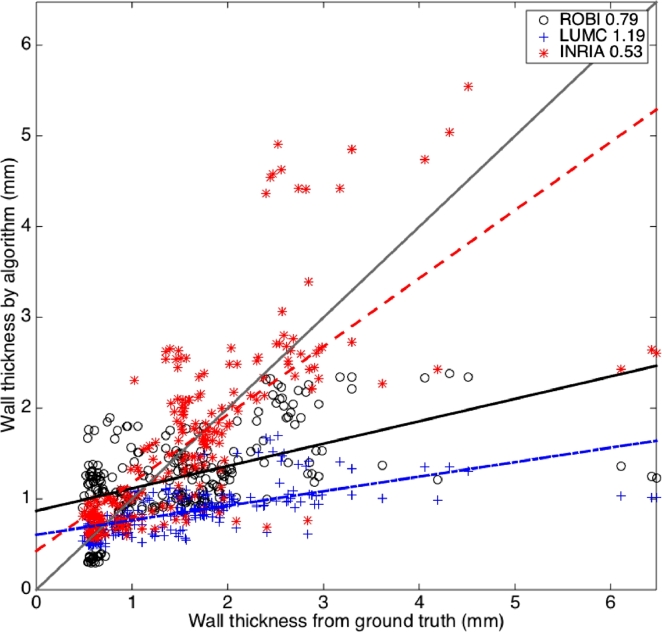
Correlation between LAWT from ground truth and algorithm segmentations over several selected slices (n=237) from images in the database. The MSE (in mm) is noted for each algorithm.

It was also possible to visualise these accuracy trends in Fig. 12 and for all images combined irrespective of quality in Fig. 13. This permitted a more objective evaluation. Clearly some algorithms suffered more than others. For example, ROBI attained a negative gradient of its straight-line fit signifying more randomness in poor quality scans.

### 3.6 Algorithm performance under artefacts

**Fig. 14:**
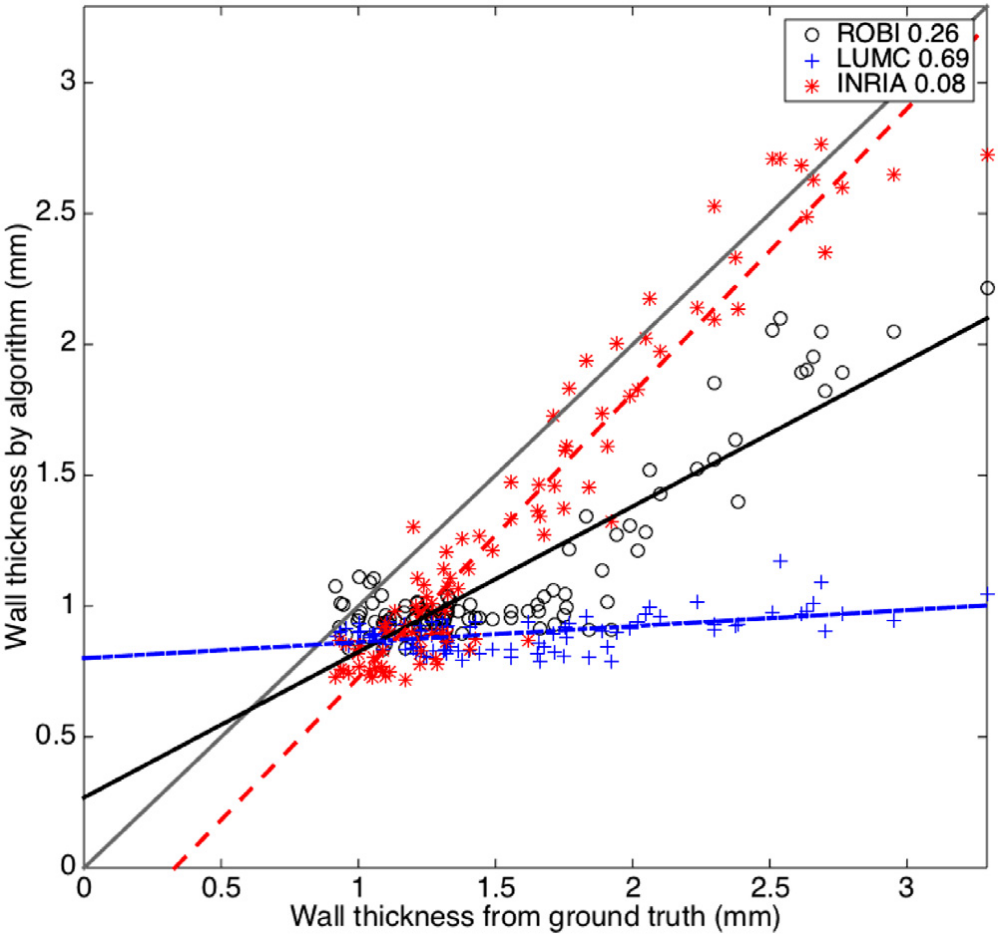
Correlation between LAWT from ground truth and algorithm segmentations on slices containing CRT lead artefact (n=97). The MSE (in mm) is noted for each algorithm.

The algorithms were also objectively evaluated under the presence of commonly found artefacts in CT cardiac scans. Slices with artefacts from pacing lead wires were selectively chosen (n=97). Streak artefacts from metallic lead wires impacted on measurements of LAWT. The correlation between algorithm and ground truth thickness measurements in Fig. 14 show that some algorithms (i.e. LUMC) were impacted more than others (i.e. INRIA). However, it was observed that the goodness of fit in these selected slices were similar to the ones encountered for all images. The selected slices with artefact was also generally a good quality image with less noise. Sections not affected by the artefact produced decent correlation. As a result, streak artefacts had minimal effect on the average thickness along a slice, as thickness values from other parts of the image would eventually smooth it out.

### 3.7 Mean thickness atlas

**Fig. 15:**
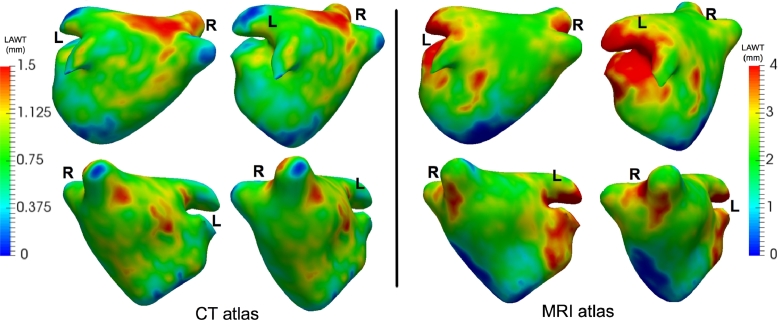
The mean thickness atlas on a 4-vein anatomical mean shape of the left atrium shown in four different orientations. The mean thickness was obtained from the consensus ground truth on all images from the database.

**Fig. 16:**
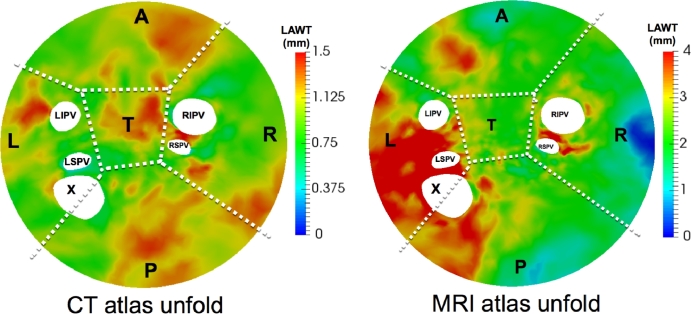
Flat 2D compact representations of the thickness atlas *unfolded* showing sections as indicated by labels L (left), R (right), A (anterior), P (posterior), T (roof) and X (appendage). The five circular holes in the map represent left inferior pulmonary vein (LIPV), left superior pulmonary vein (LSPV), right superior pulmonary vein (RSPV) and right inferior pulmonary vein (RIPV).

**Fig. 17:**
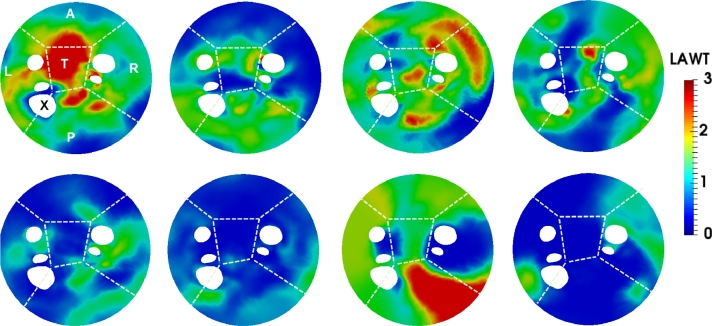
Flat 2D compact representations of thickness maps of eight images from the CT database to allow standardised comparison. In each flat map, the five holes represent four pulmonary veins and the left atrial appendage marked with an X. Each map is divided into separate sections as indicated by labels L (left), R (right), A (anterior), P (posterior), T (roof).

The mean LAWT atlas was computed by registering all patient-specific surface meshes to a 4-vein anatomical mean shape and propagating the patient-specific LAWT to the mean shape using a nearest neighbour search. Once the patient-specific LAWTs were in a common co-ordinate frame, they were averaged over all images on the database. The mean LAWT atlas can be seen in Fig. 15. It was also unfolded in Fig. 16 to a 2D flat fixed circular template so all sections could be visualised on a single plane. The thickness map from each case was also unfolded so it could be compared in 8 CT images as shown in Fig. 17. Regional wall thickness variations within the atlas are given in Table 9.

**Table 9:** Regional thicknesses with standard errors in millimetres from atlas of CT and MRI dataset. Abbreviations: Post - posterior, Ant - anterior.

	Ant.	Post.	Left	Right	Roof
**CT**	0.80 (0.21)	0.92 (0.18)	0.66 (0.19)	0.73 (0.16)	0.99 (0.28)
**MRI**	2.04 (0.72)	2.30 (0.67)	3.34 (0.86)	1.80 (1.03)	2.38 (0.37)

The mean LAWT atlas was compared to thickness reported in the literature. It should be noted the CT atlas represented a diseased cohort and MRI atlas represented healthy subjects in a younger age group. On the anterior wall, the thickness in the atlas was CT: 0.80 ± 0.21 mm, MRI: 2.04 ± 0.72 mm, compared to Pan et al. (2008) where the anterior wall was reported to be 2.0 ± 0.9 mm in the 40 to 50 age cohort. Comparing to some other studies: in Beinart et al. (2011), the reported values were 2.15 ± 0.47 mm for the mid roof (centre of the anterior region for this study) and in Hayashi et al. (2014) the reported values were 2.20 ± 0.51 mm for middle roof (centre of anterior region), all using the CT imaging modality. In the atlas, the roof was measured to be 0.99 ± 0.28 mm.

On the posterior wall, the thickness in the atlas was CT: 0.92 ± 0.18 mm, MRI: 2.30 ± 0.67 mm. This was compared to values reported in Pan et al. (2008): 1.1 ± 0.3 mm for 40–50 years old, 1.5 ± 0.3 mm for 50–60, 1.8 ± 0.2 mm for 60–70, and 1.9 ± 0.2 mm for 70–80. Pan et al. (2008) reported a difference between the anterior and posterior walls, and in the atlas there was a difference between these walls (two-tailed *t*-test with unequal variance *p* < 0.001), although they may not be directly comparable as the former did not utilise an atlas. Also, in the atlas the posterior-superior thickness for CT was 1.04 ± 0.15 mm and posterior-inferior was 0.87 ± 0.14 mm. Similar measurements reported in literature in Beinart et al. (2011) and Hayashi et al. (2014) found a higher thickness between 1.40 mm and 1.60 mm.

One potential application of the atlas is to propagate the atlas thickness to new cases. This was validated with a LOT cross-validation approach on the CT atlas. A total of 10 atlases were generated for all ten images from the database, excluding an image in each turn. The LOT atlas was then used to measure the LAWT at every location (i.e. surface vertex) on the image that was excluded from the atlas. The thickness derived from the LOT atlas and the actual thickness was compared with a point-by-point difference. An average of 52,000 points/vertex in each case was used in the calculation. Table 10 provides a summary of these results.

**Table 10:** Atlas thickness propagation validation using a leave-one-out (LOT) cross-validation. A comparison by looking at the differences between wall thickness derived from the LOT atlas and actual thickness from ground truth. The number of points (i.e. surface vertices) used in the calculation is specified (1k = 1000).

Case	Mean difference (mm)	Total points
**1**	0.76	21k
**2**	0.51	69k
**3**	0.63	76k
**4**	0.58	61k
**5**	0.52	111k
**6**	0.79	34k
**7**	1.52	22k
**8**	0.86	21k
**9**	0.58	34k
**10**	0.61	69k
**Median**	0.74	47k
**Mean**	0.62	51k

## 4 Discussion

With this atrial wall challenge, we provided a publicly available dataset with a fair and independent evaluation system. It evaluated the state-of-the-art in segmentation of atrial wall for thickness from CT and MRI. Evaluating the performance of these algorithms will provide a benchmark for future developments in this topic, which is becoming increasingly relevant in image-guided cardiac interventions. Based on the results obtained from the challenge, we are also able to provide well-founded recommendations for future developments. In this section, we will discuss insights from results, outcomes of the challenge and answer some of the questions this challenge had been designed to find out about.

### 4.1 Rankings and accuracy

We firstly consider whether the task of segmenting atrial wall still remains a challenge for computer algorithms. Based on the analysis of inter-rater differences, human raters were found to be more superior (e.g. Dice = 67) than the top-ranked algorithm (e.g. Dice = 43) from this challenge. The thin structure of the wall, generally to be under 2 mm, compared to the imaging (0.4–1 mm) resolution available makes the segmentation a very difficult task. Low Dice scores (i.e. 40–60) supports this observation. Previously published works on similar segmentation tasks such as thin lesions in brain (Maier et al., 2017) have obtained average Dice scores of 60.

LUMC came top-ranked and its Dice scores were statistically better than INRIA and ROBI. But, it was only slightly better than INRIA overall. No clear winner was found in CT. However, in MRI, level-set was a clear winner over the other techniques tested. The challenge utilised a ranking schema that computed the average final score based on ranks obtained within each case and metric. Future algorithms can be ranked fairly based on this schema that ranks on all three metrics. There remains room for further algorithm improvement and the scope for making it better could be by cross-comparing future algorithms on publicly-available benchmarked dataset rather than private image sets.

### 4.2 Insights into algorithms

A majority of the evaluated algorithms in both CT and MRI estimated the wall from the endocardial segmentation. The endocardial segmentation was extended further using a level-set or active contour. Active contour models are quite suitable in this setting as they incorporate shape constraints and it seemed to be a common theme, with both LUMC and INRIA employing it at some stage in their process. A common issue was leakage into surrounding tissue such as the neighbouring aortic wall.

Upon further investigating Leakage, the Dice and volume difference metric did not provide much insight. However, the thickness metric provided more insight and by studying the thickness correlation (Figs. 12 and 13) it was possible to look more objectively at leakage. The upper left corner of these scatter correlation plots contained points (i.e. slices) where thickness was over-estimated due to leakage. Generally, on good quality scans, there was little leakage in LUMC and INRIA, except for ROBI which leaked heavily for very thin sections of wall. In the poor quality scans, leakage became slightly more problematic, but overall top-ranked LUMC rarely leaked and it used active contours with shape constraints. In MRI, there was leakage in both region growing and watershed segmentations. Region growing over-estimated thickness in most cases and fared poorly in all segmentations. The standard level-set approach rarely leaked and had significantly better correlation with ground truth than others (Mann–Whitney test *p* < 0.05).

Future algorithms could exploit neighbouring tissue interfaces for better accuracy. Appearance models of neighbouring tissue and models of tissue-tissue interfaces should be studied in more detail.

### 4.3 Participation and importance

There was no participation in MRI and few participants for CT. However, a large number of institutions had expressed interest for the data, but did not reach submission stage. Atrial wall segmentation is generally a difficult task and CT techniques cannot easily be applied to MRI and vice versa. It is an important problem as the atria is thin structure and confounding measurements are reported in literature. Previous works made simple measurements from discrete locations of atria in imaging, and few recent works have made complex calculations for obtaining a reliable measure of thickness (Bishop, Rajani, Plank, Gaddum, Carr-White, Wright, O’neill, Niederer, 2015, Varela, Morgan, Theron, Dillon-Murphy, Chubb, Whitaker, Henningsson, Aljabar, Schaeffter, Kolbitsch, et al., 2017). The benchmark will provide a framework for future development and improve accuracy of measuring techniques.

Some of the algorithms evaluated are close to clinical use. LUMC and ROBI were both used to measure thickness in other diseased cohorts (Tao, Milles, Zeppenfeld, Lamb, Bax, Reiber, van der Geest, 2010, Inoue, Skanes, White, Rajchl, Drangova, 2014). However, further work will be needed to increase their robustness to overcome the variety of confounding factors that commonly appear in clinical practice.

### 4.4 CT and MRI comparison

The thickness measured from CT and MRI differed, where CT was consistently lower than MRI. They measured different cohorts (i.e. diseased and healthy) from different age groups. The CT cohort was expected to have thicker walls as the subjects were an older group with cardiac diseases. However, with the CT resolution being double that of MRI, there were obvious advantages to measuring a thin wall with a high resolution. Previous works have reported CT to have lower thickness than histology (Becker, 2004). The regional thickness variation in Table 9 highlights the difference between modalities.

It could be said that the MRI data was generally harder to segment than CT. The inter-rater agreement overall was lower in MRI than CT. However, the top algorithm in CT was less accurate than the top algorithm in MRI. But, overall, algorithms were more accurate in CT. It was not a like-for-like comparison as state-of-the-art algorithms in MRI were simply not available.

In MRI, the greatest variation was found in the fundi of the left atrial appendage and this region also had the most inter-subject morphological variability. Also, in MRI it was not possible in some slices to distinguish the left atrium wall and the aortic root wall. The entire border between the LA and the aortic root was included, as introducing a separation in this area would be highly subjective.

### 4.5 Wall thickness for clinical diagnosis

Previous works report thickness at various sites as there is clinical motivation to understand thickness variations between regions (Pan, Tsao, Chang, Chen, Chen, 2008, Platonov, Ivanov, Ho, Mitrofanova, 2008). Moreover, fine-grain analysis of thickness has value in clinical diagnosis. The measurement of the proportion or thickness of healthy viable tissue in the ventricle for potent areas of revascularisation is envisaged to be in the next-generation of cardiac catherisation procedures (Behar et al., 2017).

Single mean values of thickness could be more useful for population-based studies. This makes easier comparison of variation in wall thickness with patient factors such as demography or lifestyle. One of the aims of the challenge was to develop methods that automatically make dense measurements such that obtaining a reliable value for the mean thickness could be possible.

### 4.6 Image quality and artefacts

Objective evaluation based on image quality clearly demonstrated degradation of algorithm accuracy. Cross-correlation coefficients of the fit between actual and measured values were obtained to statistically verify this trend (see Table 8). Cardiac scans generally can vary with quality. The images collected for this challenge needed reliable ground truth data and poorer scans were normally avoided.

Slices with streak artefacts due to pacemaker were also selected for evaluation. Although it was clear that image quality affected algorithm accuracy, Fig. 12, Fig. 13, Fig. 14 demonstrated that the algorithm performance was not statistically inferior than their performance over all slices. For example ROBI’s ρ=0.96 in artefact versus ROBI’s ρ=0.73 over all images, with similar trend in LUMC. Slices with streak artefacts generally affected a small portion of the wall and its effects were smoothed out by the remaining portion. Streak artefacts, in our small study of n=97 slices, had a minimal effect on accuracy as thickness could be reliably derived from other sections of the wall.

### 4.7 Limitations

The proposed work has several limitations. An important limitation is the image database size (n=20). Within this small sample size, to mitigate this limitation, a large data pool was generated with several hundreds of slices per datasets, resulting in thousands of data points. The algorithms could be compared using point-by-point data analysis on several tens of thousands of individual locations resulting in high-density measurements for comparison. To our knowledge, the scale of this analysis on LA wall imaging data is novel as most previous work has relied on sparse measurements made at few selected areas.

A second limitation is the method in which thickness is calculated in this work. The thickness was determined as the shortest Euclidean distance from the outer to the inner boundary of the segmented wall (refer to Eq. (4)). However, this method can give spurious lengths in instances where the wall is thicker and has a sharp corner. In Bishop et al. (2016) these situations are addressed and they proposed a Laplace equation used in Electromagnetism to construct field lines to solve for thickness. The method is slow due to its finite element method approach. The spurious lengths in thicker and sharp corners can be expected to have a negligible effect in our calculations as these occurrences constitute less than 5% of our data. When they do occur at some locations on the wall, data from some individual pixels are affected and its effect on the overall slice average is negligible.

## 5 Conclusions

This work proposes an open-source benchmarking dataset for left atrial wall segmentation algorithms. Left atrial wall segmentation is currently a relevant and important problem as recent studies have shown that treatments for AFib are highly dependent on the success of creating contiguous transmural lesions on the left atrial wall. CT is the optimal modality for imaging the wall and MRI images the wall non-invasively. Algorithms that segment the wall from CT and MRI are few. It is not clear how algorithms compare or perform relative to one another. Three published techniques for wall segmentation were validated and benchmarked in this work, and three standard image processing techniques for MRI. The translation of future algorithms into the clinical environment becomes challenging if they are only tested on centre-specific private image repositories. The proposed work provides a publicly-available dataset of twenty images and evaluation strategies such that wall segmentation algorithms can be compared on a benchmark. The work is timely as more algorithms are expected to be written in future and their comparison can become difficult. The proposed benchmarking dataset remains publicly available for accessing the image database. The datasets are now publicly available via the website at: http://stacom.cardiacatlas.org.

## Conflict of interest

The authors declare no conflict of interest.
